# Voices of Survivorship: The Unmet Needs of Italian Cancer Survivors. A Qualitative Study

**DOI:** 10.1002/cam4.71121

**Published:** 2025-08-01

**Authors:** Angela Contri, Stefania Costi, Monica Guberti, Silvia Soncini, Stefano Botti, Andrea Frasoldati, Martina Torreggiani, Luca Ghirotto

**Affiliations:** ^1^ Clinical and Experimental Medicine PhD Program University of Modena and Reggio Emilia Reggio Emilia Italy; ^2^ Physical Medicine and Rehabilitation Unit Azienda USL‐IRCCS di Reggio Emilia Reggio Emilia Italy; ^3^ Department of Surgical, Medical, Dental and Morphological Sciences Related to Transplant, Oncology and Regenerative Medicine University of Modena and Reggio Emilia Modena Italy; ^4^ Research and EBP Unit Azienda USL‐IRCCS di Reggio Emilia Reggio Emilia Italy; ^5^ Hematology Unit Azienda USL‐IRCCS di Reggio Emilia Reggio Emilia Italy; ^6^ Endocrinology Unit Azienda USL‐IRCCS di Reggio Emilia Reggio Emilia Italy; ^7^ Qualitative Research Unit Azienda USL‐IRCCS di Reggio Emilia Reggio Emilia Italy

**Keywords:** breast cancer, focus groups, framework analysis, lymphoma, myeloma, prostate cancer, qualitative, survivorship, thyroid cancer, unmet needs

## Abstract

**Background:**

The increasing number of cancer survivors (CSs) globally highlights the critical need for healthcare systems to address their unmet needs. These needs span physical, psychosocial, spiritual, informational, and practical dimensions and, if unaddressed, can impact quality of life and healthcare satisfaction.

**Aims:**

This study aimed to identify and understand the unmet needs of CSs in Italy to guide the development of patient‐centered survivorship care services.

**Methods:**

A cross‐sectional qualitative study was conducted between April 2023 and January 2024 at the Comprehensive Cancer Centre of Reggio Emilia, Italy. Data were collected through focus groups and individual interviews with 35 CSs and seven caregivers selected via convenience sampling. Eligibility criteria included non‐cutaneous CSs with a 5‐year survival rate of ≥ 65% who had completed active treatment and were in follow‐up care. Data were analyzed using the Framework Method.

**Results:**

Four key themes emerged: (1) Dignity and Respect: Emphasis on the importance of treating CSs with dignity and respect within healthcare settings; (2) Desire for Normality: Highlighting CSs' strong desire to regain a sense of normalcy post‐treatment; (3) Pursuit of Control Over One's Life: CSs' need to maintain control, particularly regarding information needs and treatment management; (4) Existential Vulnerability: The vulnerability and fragility felt by CSs, underscoring their need for emotional support and reassurance.

**Conclusions:**

Unmet needs remain a significant challenge for CSs, necessitating the implementation of tailored, patient‐centered care interventions. Addressing these needs can enhance quality of life, satisfaction, and outcomes for CSs worldwide.

**Trial Registration:** ClinicalTrials.gov Identifier: NCT06236373

## Graphical abstract

Beyond medical survival, cancer survivors in Italy reveal a nuanced landscape of unmet emotional and existential needs, challenging conventional post‐treatment care models. This study illuminates the significant discrepancy between the clinical outcomes and the lived experiences of those affected by cancer, advocating for a more holistic approach to survivorship that centers on personal dignity and agency.

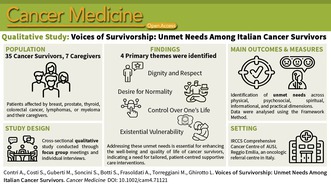

## 1 Introduction

The increasing number of cancer survivors (CSs) worldwide emphasizes the importance of providing services that meet their specific unmet needs [1].

Individuals are identified as ‘cancer survivors’ from the moment of diagnosis and throughout their lifetime [2]. It is widely acknowledged that CSs face numerous challenges across physical, psychosocial, spiritual, informational, and practical dimensions [3, 4, 5, 6]. Despite this recognition, these challenges often lead to unmet needs not adequately addressed by healthcare systems [7].

Within this framework, ‘unmet needs’ denotes the deficiencies individuals perceive in the level of service required to attain optimal well‐being [8]. These needs are defined by unmet demands that prompt CSs to seek additional assistance or support [9]. Thus, a comprehensive understanding and evaluation of the unmet needs experienced by CSs are paramount to pinpointing discrepancies in their care experiences and to facilitating the delivery of patient‐centered services [10]. Effective, patient‐centered care delivery can improve patient outcomes, quality of life, and satisfaction with care [11] and may lead to a reduced demand for health and social care services [12].

Identifying unmet needs must begin with listening to the actual voices of individuals experiencing a condition and to their caregivers. We focused on defining functional domains that matter to CSs to ensure a thorough understanding of their challenges and to facilitate the development of targeted interventions and supportive services that address their specific needs.

This approach acknowledges cancer survivorship as a complex phenomenon that includes the physiological effects of the disease and its treatments as well as any and all emotional, social, and practical consequences. Including patients and caregivers, both with experience of cancer, facilitated a comprehensive exploration of the challenges and requirements encountered by css.

## 2 Methods

### 2.1 Study Design

A cross‐sectional qualitative consensus‐based study was conducted from April 2023 to January 2024. The study used focus group meetings (FGMs) and individual interviews. The Consolidated Criteria for Reporting Qualitative Research (COREQ) were followed for reporting [13, 14].

The protocol of this study was registered on ClinicalTrials.gov (ID NCT06236373).

### 2.2 Participant Involvement

Participants were recruited from the Comprehensive Cancer Centre of Reggio Emilia, a center of excellence in cancer research serving an average of 9000 cancer patients per year.

Patient participants, chosen based on their clinical diagnosis, met the following inclusion criteria:

– Having received a cancer diagnosis (non‐cutaneous) with a 5‐year survival rate ≥ 65%, i.e., breast, prostate, thyroid, colorectal cancer, lymphomas, and early‐stage multiple myeloma.
– Having completed the active phase of treatments and in follow‐up at the Comprehensive Cancer Centre of Reggio Emilia.

Patients were excluded if they were aged < 18 years or presented with comorbidities that could have hindered their participation in the study (e.g., cognitive limitations).

Caregiver participants were invited to participate if identified as caregivers by the contacted patients. Caregivers with cancer were excluded.

### 2.3 Focus Group and Interview Guide Development

After reviewing the literature on the main dimensions, topics, and questions included in patient‐reported outcome measures (PROMs) used internationally to identify the unmet needs of CSs, we identified a list of themes to gain a holistic view of all possible unmet needs that our CSs may have experienced. These themes were used to develop preparatory material to be sent to participants before the FGM/interview took place.

The subsequent FGMs and interviews were guided first by reflecting on the preparatory material, then by using the most relevant aspects that emerged to stimulate discussions. Participants were also asked to identify important aspects not covered in the material.

### 2.4 Data Collection

We conducted FGMs and qualitative interviews involving patients and caregivers navigating their cancer journey. FGMs represent a data collection method that enables qualitative researchers to efficiently gather insights from multiple participants concurrently. A more dynamic and insightful dialog can ensue through collective discussion within a group. The moderator facilitates the focus group to encourage an open exchange of ideas, thus yielding valuable data from a specific population on a particular area of interest. FGMs offer a more relaxed setting than do one‐on‐one interviews, thereby fostering the feeling among participants that they can freely articulate their perspectives in the presence of others. Furthermore, FGMs allow participants to exchange ideas, which can potentially lead to uncovering diverse viewpoints during the discussion [15].

Individual interviews were organized for those participants who could not attend the FGMs for logistical reasons. These interviews covered the same topics as the FGMs and were conducted online or in person.

### 2.5 FGM Organization

In line with recommendations for conducting qualitative research in healthcare settings [16], patient participants with the same cancer diagnosis took part in the same FGM, which consisted of three to eight participants [17]. This ensured that the participants' experiences would be similar enough for them to feel comfortable enough to openly and honestly share the practical, physical, emotional, spiritual, and informational issues they may have been facing as CSs and that could lead to “unmet needs.”

The preparatory material for the qualitative data collection phase was sent to each participant approximately 1 week before the FGM/interview to allow for enough time to read it.

This material consisted of 25 questions derived from the PROMs developed and used to identify the unmet needs of CSs worldwide [18]. The aim of these questions was as a warm‐up exercise to begin thinking about the themes that would guide the subsequent discussions. The preparatory material is attached as Appendix A.

The interviews/FGMs with the participants were audio recorded, and the audio file was canceled immediately after its verbatim anonymized transcription was completed by a specialized professional studio (See Appendix B). Transcripts were not returned to participants.

### 2.6 Data Analyses

Sociodemographic data and disease‐related factors were analyzed using descriptive statistics.

Data from the FGMs and the interviews were analyzed using an inductive/deductive framework. The Framework Method has been widely and successfully used in research for over 35 years [19] and has recently gained popularity as an analysis method in qualitative health research [20]. This method provides a structured, clear approach to summarizing data, making it particularly valuable for multidisciplinary research teams where not all members have experience in qualitative data analysis, as in this case. It facilitates a comprehensive and descriptive overview of the entire dataset, even when dealing with large amounts of data.

An inductive framework guided data processing from the preparatory material, while a deductive framework guided the analysis of data gathered during interviews and FGMs.

Two researchers (AC, SC) carried out this process independently, and a third researcher (LG) helped resolve discrepancies.

**FIGURE 1:**
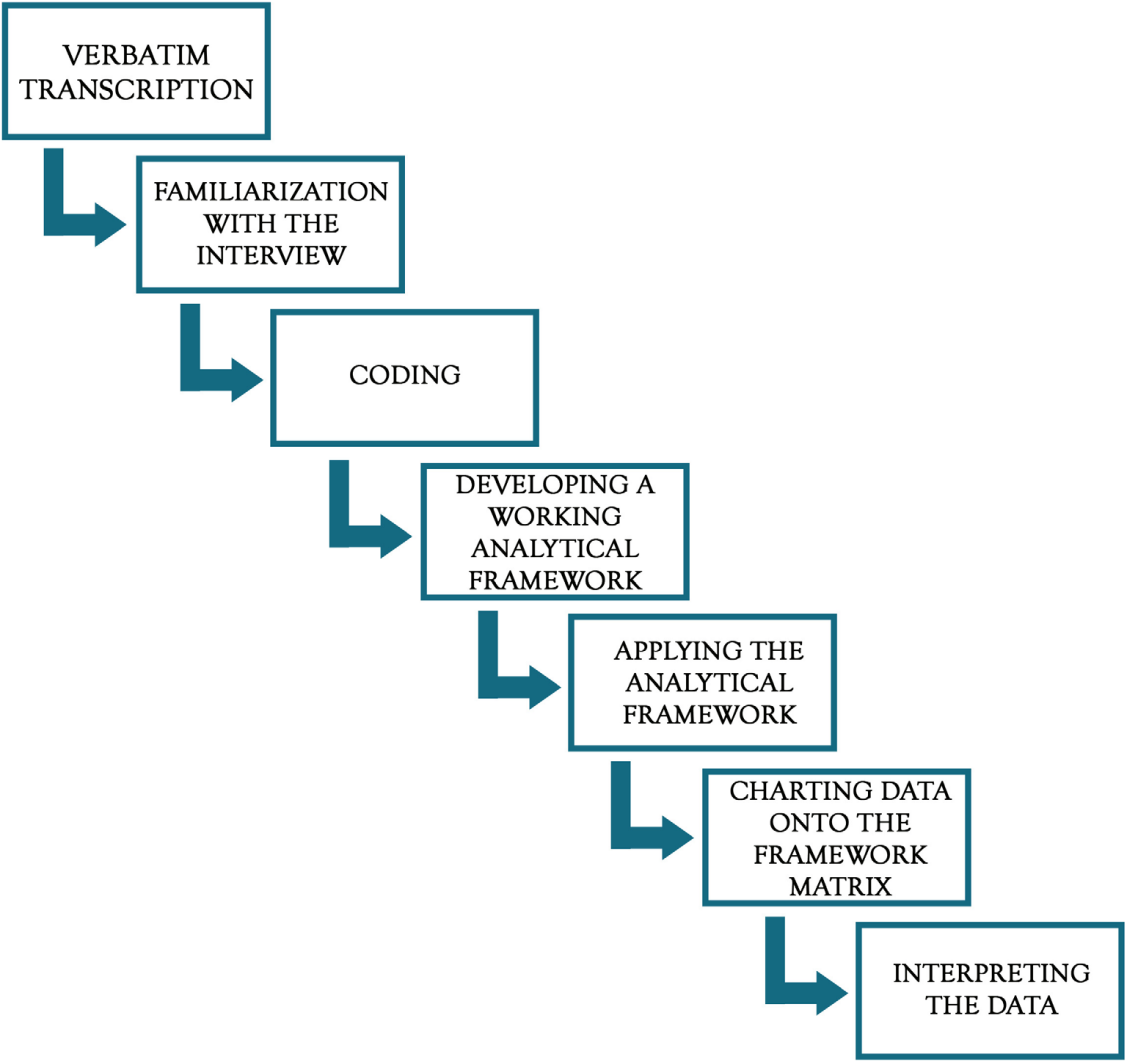
Framework analysis flowchart.

The seven steps of framework analysis, presented in Figure 1, were followed sequentially.

Details of the data analysis methodology are reported in Appendices B, C, D.

### 2.7 Ethical Considerations

The study's protocol was approved by the Ethics Committee, Area Vasta Emilia Nord (in‐house protocol n. IRCCS_RE_CSSC_01), and written informed consent was obtained from all participants prior to study enrollment.

## 3 Results

In this study, we used semi‐structured interviews and FGMs to collect data from 42 participants, including 35 patients who had been diagnosed with non‐cutaneous cancer and who had completed the active phase of treatments, as well as seven of their caregivers.

Twenty other potential patient participants were excluded because they did not consent to take part (29%), did not respond to contact attempts (N. 10%, 14%), or did not show up at the appointment (N. 5%, 0.7%), thus resulting in a participation rate of 50%.

We conducted eight FGMs (six with patients, two with caregivers) and nine individual interviews.

Details about each FGM are provided in Appendix E.

The average duration of each FGM was 86 min (range: 67–114).

Three individual interviews were conducted with patients diagnosed with prostate cancer, five with patients diagnosed with thyroid cancer, and one with a patient diagnosed with lymphoma.

The individual interviews lasted an average of 58 min (range: 42–95).

Data saturation was reached after four FGMs.

FGMs and in‐person individual interviews were held at the AUSL‐IRCCS of Reggio Emilia healthcare facilities in the presence of only the participants and the investigators.

Online interviews were conducted using the Google Meet video conferencing app.

### 3.1 Participants' Characteristics

Participants' Characteristics Are Reported in Table 1.

**TABLE 1:** Participants' characteristics.

Status	Type of cancer	N	Mean Age (SD)	Sex	Living	Education level	Profession	Mean months elapsed since the end of the therapies
Pt	Prostate	7	73.1 (±6.4)	M (100%)	Alone (14.3%) With a partner (71.4%) With family (14.3%)	Mandatory schooling (50%) University degree (50%)	Office worker (14.3%) Retired (85.7%)	16.0 (±7.8)
Pt	Breast	9	59.6 (±4.1)	F (100%)	With child/children (22.2%) With family (33.3%) With a partner (44.4%)	High school diploma (44.4%) University degree (55.6%)	Teacher (22.2%) Physiotherapist (11.1%) Office worker (22.2%) Nurse (11.1%) Retired (22.2%) Self‐employed (11.1%)	13.8 (±13.0%)
Pt	Lymphoma	4	59.0 (±4.7)	M (25%); F (75%)	With family (50%) With a partner (50%)	Mandatory schooling (75%) High school diploma (25%)	Bricklayer (25%) Metalworker (25%) Unemployed (25%) Nurse (25%)	12.3 (±10.9)
Pt	Myeloma	5	61.8 (±6.8)	M (40%); F (60%)	With a partner (80%) With family (20%)	Mandatory Schooling (60%) High school diploma ($0%)	Supermarket cashier (20%) Butcher (20%) Housewife (20%) Retired (40%)	Maintenance therapies ongoing
Pt	Colorectal	5	59.4 (±7.7)	M (60%); F (40%)	With a partner (80%) With child/children (20%)	Mandatory schooling (20%) University degree (40%) High school diploma (40%)	Baker (20%) Teacher (20%) Retired (20%) Surveyor (20%) Artisan (20%)	44.6 (±14.1)
Pt	Thyroid	5	40.8 (±9.2)	M (20%); F (80%)	Alone (40%) With family (40%) With a partner (20%)	University Degree (60%) High school diploma (40%)	Chemist (20%) Teacher (20%) Student (20%) Office worker (20%) Metalworker (20%)	42.6 (±48.6)
**Status**	**Type of cancer**	**N**	**Mean Age (SD)**	**Sex**	**Relation with CS**			
Cg	Prostate	2	61.5 (±3.5)	F (100%)	Wife (100%)			
Cg	Thyroid	2	66.5 (±5.5)	F (100%)	Mother (100%)			
Cg	Breast	1	73	M (100%)	Husband (100%)			
Cg	Colorectal	2	68.5 (±3.5)	F (100%)	Wife (100%)			

Abbreviations: Cg, caregiver; F, female; M, male; Pt, patient; SD, standard deviation.

The CSs had a mean age of 58.8 years (±11.4), 21 (60%) were female, and 32 (91.4%) were living with a partner or family. Eleven (31.4%) were retired, while the others were employed (22, 62.6%), students (1, 2.9%), or unemployed (2, 5.7%). The months elapsed since the end of treatment ranged from 0 to 108 three participants (8.6%) were still on maintenance therapy (mean duration: 23.5 (±27.2) months).

The caregivers were primarily women (6, 85.7%) and included four wives and two mothers as well as one husband. They cared for patients diagnosed with prostate (two, 28.6%), thyroid (two, 28.6%), colorectal (two, 28.6%), and breast (one, 14.3%) cancer. Of these caregivers, five (71.4%) were related to a CS who attended one of the patients' FGM.

### 3.2 Findings

We identified four primary themes, encompassing 16 subthemes. These main themes were ‘dignity and respect’, ‘desire for normality’, ‘pursuit of control over one's life’, and ‘existential vulnerability’ (Table 2), which together encapsulated the underlying meaning of the unmet needs expressed by the participants.

**TABLE 2:** Main findings.

Primary themes	Findings	Meaningful quotations	Individual codes	Topic areas	
				Subareas	
Dignity and respect	The concept of having one's dignity acknowledged and receiving respect from others was highlighted by CSs when discussing the emergence of unmet needs related to health and support services. Specifically, they expressed the need for access to benefits and the right to use services related to their condition to be treated with greater respect during their illness without feeling like a burden on family or society, and the need for health services specifically tailored to their needs. They deemed important feeling respected as individuals and being accompanied throughout their treatment with their dignity intact. Caregivers also expressed a similar sense of urgency.	“What pisses me off is that I have to book my mammogram the same way as all other women who are not former cancer patients; so we're on the general appointment calendar, we don't have a preferential, priority route when we enter follow‐up, the famous follow‐up…” Breast CS. “This stuff pisses me off a bit, I can tell you… because…I have an exemption that is only valid for five years, but it's not like my thyroid will grow back in five years… …The fact that I need certain drugs and every time I have to go to the doctor for a prescription, for me it's a hassle. I mean, it's not that you go back there, it's a drug that I will only need for a certain amount of time, I mean… I'll need it forever! So, I sincerely don't understand why if I go to the pharmacy with my little piece of paper telling me that I need thyroid medication for life and I have this exemption, why I have to go to the doctor every time to renew it…” Thyroid CS	Need for benefits and rights to benefit from services related to my condition	Needs related to health and support services	Needs related to health and support services (information, support…)
			Need to be able to have health services suited to my needs (in quality and quantity)		
			Need to be able to have appointments, consultations, tests, and therapies available when needed		
			Need for support services for housework and transportation		
			Need to receive clear information about personal rights	Need for Information	
			Need to manage one's own finances	Need to manage one's own finances	
			Need to manage the work‐school environment	Need to manage the work‐school environment	
			Need for support from and relationships with healthcare professionals^a^	Need for support from and management of relationships with others (health professionals, partners, family, friends, coworkers, etc.)	
			Need for support from and relationships with others (friends, coworkers, etc.)^a^		
			Need for support from and relationships with partners, children, and family^a^		
Pursuit of control over one's life	The desire to have a sense of control over one's life emerged forcefully in the context of information needs and, in those needs, linked to the procurement and management of drugs, therapies, and their side effects.	“Radiotherapy is sold as an alternative to the surgical part, it's sold as if it was a piece of cake; it's not true…” Prostate CS “I would have liked someone to talk to who could explain it well, who didn't use technical terms, which I couldn't remember by heart at the time, and then I read it, but then I had to, I went home, I went to look it up…” Breast CS “You have to find the right type of diaper; here, for example, nobody gives you any tips, what brands there are, how tight they hold, you have to experiment…” Prostate CS “Regarding impotence… no one had spoken to me about it…” Prostate CS “I would have been more pleased to know what was going to happen to me next…” Colorectal CS	Need for a support service to turn to that will clarify all one's doubts about the therapeutic pathway	Needs related to health and support services (information, support…)	Needs related to health and support services
			Need for more and clearer information about one’ current health status		Need for Information
			Need information on available services		
			Need more information about medical devices (e.g., for incontinence) and their use		
			Need for more information on how to aid one's recovery		
			Need more information on possible therapeutic pathways		
			Need to receive more information about the purpose and effects of therapies		
			Need to know who to turn to in case of need		
			Need to manage expectations (one's own and others')	Needs related to the management of emotional‐psychological aspects	
			Need for emotional support		
			Need to manage side effects of medications or therapies	Need to manage drugs, aids, therapies, and their side effects	
			Need to procure and manage the intake of drugs, aids, or therapies		
			Management of one's identity and oneself	Need to manage one's identity and self	
			Need to manage substance use/abuse	Need to manage substance use/abuse	
			Need to manage nutrition and weight control	Need to manage basic bodily functions (sleep, nutrition, bowel, bladder…)	
			Need for independence in urban mobility	Need for independence in urban mobility	
			Need for support from and relationships with healthcare professionals^a^	Need for support and management of relationships with others (health professionals, partners, family, friends, coworkers, etc.)	
			Need for support from and relationships with others (friends, coworkers, etc.)^a^		
			Need for support from and relationships with partners, children, and family^a^		
Existential vulnerability	During the FGMs with cancer CSs, another aspect was strongly emphasized. The need not to feel alone, to be told that everything will be fine, to have access to inclusive services not only for oneself but also for one's entire family sphere, services that support one's overall well‐being.	“I had to make the decision myself, they wouldn't let my wife in…” Prostate CS “What don't I have, what am I missing at this moment in my life? The support of healthcare, which should have been there…which has forgotten about us….” Breast CS	Need for healthcare professionals who communicate more with each other	Needs related to health and support services (information, support…)	Needs related to health and support services
			Need for services that include the entire family		
			Need for inpatient and outpatient services that support one's overall well‐being		
			Need to be reassured about one's worries	Needs related to the management of emotional‐psychological aspects	
			Needs related to physical exercise	Need to manage aspects related to moving and physical exercise	
			Need to manage sleep	Need to manage basic bodily functions (sleep, nutrition, bowel, bladder…)	
			Need to realize one's spiritual needs	Need to realize one's spiritual needs	
			Need for support from and relationships with healthcare professionals^a^	Need for support and management of relationships with others (health professionals, partners, family, friends, coworkers, etc.)	
			Need for support from and relationships with others (friends, coworkers, etc.)^a^		
			Need for support from and relationships with partners, children, and family^a^		
Desire for normality	The desire for normality was strongly expressed by patients who wished to regain control over the routine of their lives. This included managing their sexual health, basic bodily functions such as bladder control or intestinal problems, and resuming their previous relationships with friends, coworkers, and family.	“It's still a mutilated body…” Breast CS “It weighs on me. It weighs on me because I liked it… Afterwards, I also said it to the doctor: in bed I dream about it sometimes… I dream about having a little orgasm…” Prostate CS “You lose all desire because your head is somewhere else…” Myeloma CS “I have days when I go to the bathroom more often, I work, so this sometimes makes me uncomfortable…. … I have a little bit more uncertainty because I can't manage my time when and how I want, so I have the problem…” Colorectal CS	Need for positive thoughts and lightness	Needs related to the management of emotional‐psychological aspects	
			Need to rationalize the fear of recurrence		
			Need to switch off thoughts		
			Needs related to movement problems	Need to manage aspects related to moving and physical exercise	
			Need to manage pain	Need to manage pain	
			Need to manage lack of energy, strength, or desire to do things	Need to manage lack of energy, strength or desire to do things	
			Need to manage memory or concentration	Need to manage memory or concentration	
			Feeling ‘normal’	Feeling ‘normal’	
			Need to manage sexuality	Need to manage the sexual sphere	
			Need to manage intestinal problems	Need to manage basic bodily functions (sleep, nutrition, bowel, bladder, etc.)	
			Need to manage bladder		
			Need for support from and relationships with healthcare professionals^a^	Need for support from and management of relationships with others (health professionals, partners, family, friends, coworkers, etc.)	
			Need for support from and relationships with others (friends, coworkers, etc.)^a^		
			Need for support from and relationships with partners, children, and family^a^		

^a^ Transversal codes that refer to all 4 primary themes.

The theme of ‘dignity and respect’ collected data underscoring the importance of acknowledging the individual's dignity and of showing respect, particularly within the context of health and support services. CSs emphasized the importance of being treated with dignity and respect as they faced the challenges of cancer diagnosis, treatment, and its aftermath.

The theme ‘desire for normality’ collected narratives that highlighted CSs' strong desire to regain a sense of normalcy in their lives after their cancer diagnosis. This theme encompassed aspects such as managing sexual health and bodily functions and re‐establishing relationships with friends, coworkers, and family.

The theme ‘pursuit of control over one's life’ regarded CSs' profound desire to maintain a sense of control over their lives, particularly concerning information needs and the management of treatment‐related side effects.

Finally, the theme ‘existential vulnerability’ represented the data on the vulnerability and fragility participants felt after their cancer diagnosis; participants emphasized their need for emotional support, reassurance, and inclusive services for both them and their loved ones.

An analysis of these themes and a sample of related meaningful quotations are reported in Table 2.

## 4 Discussion and Implications

Being a CS involves integrating the illness experience into one's life story and finding fulfillment despite it [21, 22]. This adaptive process requires overcoming and adapting to various challenges and changes by addressing impacts on the mind and body, which ultimately empowers a CS in coping and regaining a sense of autonomy in their daily lives [23]. Recognizing and addressing CSs' and their caregivers' unmet needs is crucial to providing tailored support and overcoming barriers within healthcare services.

Survivorship transcends the binary notions of “cured” and “not cured”; it involves facing biopsychosocial challenges that may last throughout a survivor's life [24].

The four identified themes provide powerful frameworks for understanding CSs' unmet needs [25, 26].

Our participants emphasized that preserving a patient's dignity and respect is crucial to quality healthcare, which encompasses both medical treatment and compassionate care. Previous studies have highlighted the importance of maintaining personal space and privacy, respecting values, and providing moral support as key elements in upholding the dignity of cancer patients [27, 28, 29]. In other words, healthcare providers must treat patients with empathy and sensitivity, dedicating time to listening to their concerns and fears and providing necessary support, thereby enhancing their overall well‐being and recovery.

The desire for normality is a complex phenomenon that encompasses many unmet needs. Normality can be seen as an outcome (being normal), a practice (“doing normality”), and an ethical standard [30]. A study conducted a few years ago revealed that it is not uncommon for cancer patients to opt to cease cancer treatment to get back to ‘normality’ because treatment is experienced as ‘a continuation of the disease’ [31]. The relatively high rate of potential candidates who refused to participate in our study may reflect this perception among our target population. It is essential that healthcare organizations consider this; they may wish to invest more in helping patients accept that this is their new normal, recognizing that things will not necessarily return to the way they were before their diagnosis.

Other studies have emphasized the themes of the loss of self‐determination, a feeling of worthlessness brought on by the disease, and experiencing a profound intrusion into one's personal life. These themes have been linked to a sense of personal frustration [32] and to a reduction in the effectiveness of the treatment [33].

Frailty, which colored many of the reported unmet needs, impacts CSs' survival, long‐term function, and quality of life [34]. While previous studies have discussed themes such as a ‘sense of loneliness’, ‘fear of death’, and ‘cancer‐related ruminations [35, 36, 37, 38, 39], we felt it appropriate to unify these feelings and perceptions in this type of analysis under the single overarching theme ‘sense of existential fragility’.

### 4.1 Implications for Clinical Practice and Research

This study highlights the critical need for innovative approaches to address the multifaceted unmet needs of CSs. Clinically, our findings underscore the importance of adopting patient‐centered care models that not only focus on disease management but also prioritize dignity, respect, and emotional well‐being. Healthcare providers should integrate practices that promote autonomy and normalize survivorship as a unique phase of life, empowering patients to develop adaptive strategies for achieving optimal functioning in their ‘new norma’. Tailored interventions should consider the pervasive sense of existential fragility reported by CSs, implementing structured support systems to mitigate its impact on long‐term outcomes.

From a research perspective, this study provides a robust framework for future investigations into survivorship care. The four identified themes offer a foundation for developing and testing interventions that address biopsychosocial dimensions of care. Comparative studies across different cultural and healthcare settings could further elucidate the universal and context‐specific aspects of survivorship, guiding global strategies for tailored care. Moreover, longitudinal studies are needed to assess the long‐term effects of addressing unmet needs on quality of life and health outcomes, as well as the cost‐effectiveness of patient‐centered survivorship programs.

While ideal models of survivorship care remain aspirational, the frustrations expressed by participants, such as feelings of being disregarded, overwhelmed by bureaucracy, or insufficiently informed, highlight addressable gaps within the current system. These do not necessarily require structural overhauls, but rather targeted, scalable adjustments, including improved communication strategies, clearer information pathways, dedicated survivorship staff, and enhanced professional training on how to communicate effectively with vulnerable patients.

By bridging clinical practice with targeted research, we can move toward a comprehensive survivorship care paradigm that not only improves individual outcomes but also informs policies to optimize healthcare delivery for this growing population.

### 4.2 Policy Implications and Institutional Response

The findings of this study have prompted preliminary discussions within the Comprehensive Cancer Centre of Reggio Emilia, engaging clinical teams, hospital management, and patient advocacy groups. A critical emerging theme is the need to optimize existing resources by improving coordination among support services. While many services (e.g., psychosocial and occupational support, specific exercise groups) already exist, their accessibility is often hindered by fragmented communication and a lack of integrated networking among providers. This dispersion of resources risks duplicating efforts in some areas while leaving other unmet needs unaddressed. Moving forward, institutional efforts will focus on: (1) systematically mapping available services to identify redundancies and gaps, (2) strengthening collaborations between specialized organizations to promote expertise‐sharing, and (3) redesigning survivorship care pathways to prioritize patient‐centered, equitable access, particularly for long‐term side‐effect management and psychosocial support. These steps aim not only to address the unmet needs identified in this study but also to create a scalable framework for survivorship care that balances efficiency with tailored support. Future work will monitor the implementation of these strategies and their impact on patient‐reported outcomes.

### 4.3 Strengths and Limitations

The results of this study must be interpreted in light of its limitations. They represent the experiences of CSs living and receiving healthcare in Italy. Aware of this, we tried to recruit a sample of CSs from various sociodemographic backgrounds to enhance the transferability of our findings. Nonetheless, the relatively high rate of potential participants who opted not to participate in the study may have biased our results.

A notable strength of this study is that it represents the first instance of research conducted in Italy, providing evidence within our unique environment, health system, sociocultural context, and economic condition.

At least two researchers were involved in each data collection and analysis step, limiting any possible interpretive bias. The interdisciplinary nature of the research team allowed for challenging and corroborating the data and analytic processes. None of the authors had a prior relationship with any of the CSs involved in this study. All participants received the same information sheet. The involved researchers received training for all the research steps to ensure trustworthiness [40].

## 5 Conclusions

CSs' experiences are influenced by a myriad of factors, including cultural norms, healthcare systems, and socioeconomic conditions. Examining the needs and experiences of CSs in Italy through focus groups and individual interviews sheds light on the unique challenges Italian CSs face and the specific deficiencies in care within this context, thus enriching our understanding of survivorship in this country.

Rather than framing survivorship as a dichotomy between ‘cur’ and ‘failure’ our findings suggest a more nuanced approach: supporting survivors as they cope with the challenges of complex, often imperfect systems, while acknowledging the legitimacy of their frustrations. This perspective calls for humility, recognizing that while not all suffering can be eliminated, healthcare systems can and should prevent survivors from facing avoidable challenges, such as unclear communication, fragmented services, or the burden of managing care alone.

Our study contributes valuable insights to the global understanding of survivorship care. By comparing our findings with those of other studies conducted in different countries, we were able to identify common themes and areas of divergence, thus enhancing our comprehension of survivorship needs across diverse populations.

## Author Contributions

**Angela Contri:** conceptualization (equal), data curation (equal), formal analysis (equal), investigation (equal), methodology (equal), validation (equal), writing – original draft (equal), writing – review and editing (equal). **Stefania Costi:** conceptualization (equal), data curation (equal), formal analysis (equal), investigation (equal), methodology (equal), supervision (equal), writing – original draft (equal), writing – review and editing (equal). **Monica Guberti:** project administration (equal), visualization (equal). **Silvia Soncini:** visualization (equal). **Stefano Botti:** visualization (equal). **Andrea Frasoldati:** visualization (equal). **Martina Torreggiani:** project administration (equal), visualization (equal). **Luca Ghirotto:** conceptualization (equal), data curation (equal), formal analysis (equal), investigation (equal), methodology (equal), software (equal), supervision (equal), writing – original draft (equal), writing – review and editing (equal).

## Disclosure

Additional Contributions: We thank all the participants and their families. We would also like to extend our sincere gratitude to Jacqueline M. Costa for her exceptional scientific editing, professionalism, and invaluable expertise.

## Conflicts of Interest

The authors declare no conflicts of interest.

## Data Availability

Deidentified data and analytic code used for analyses will be made available to other researchers upon request to the corresponding author (stefania.costi@unimore.it).

## Appendix A Preliminary Questions (Pre‐Focus Group)

1. I felt the need for emotional support (from relatives, friends, professionals, people in the same situation as me)
2. I needed to manage physical pain
3. I did not do some things for fear of physical pain
4. I worried about my weight
5. I needed help to stop the diarrhea
6. I needed help controlling my bladder
7. The area of intimate and sexual relationships gave me problems.
8. I had problems with memory/lack of concentration
9. I did not feel accepted by others in my new me
10. I would have liked clearer information (about drugs, treatments, etc.)
11. I felt like a burden asking friends/relatives to do things for me
12. I had problems bending/lifting objects/moving/doing housework
13. I did not feel I could use transport (my car, bus, train, etc.) as freely as I wanted/needed to
14. Health services were not available when I needed them
15. It wasn't easy to get the medication I needed
16. I would have liked to be of help to my partner/family (e.g., to explain my situation to my children)
17. I was afraid that I might be fired
18. I needed to deal with financial problems
19. I've felt the need for spirituality/spiritual guidance
20. I was concerned about the lack of desire to do anything
21. I felt the need to deal with treatment side effects
22. I've overdone it with certain substances (e.g., drugs, alcohol, nicotine, medication, other)
23. I've felt lonely
24. My complaints have not been taken into consideration seriously by health professionals.
25. I would have liked clearer information about how long I would be absent from work

## Appendix B Details of the Framework Method Used

### Step 1: Verbatim Transcription

To ensure similarity in transcription style across the whole dataset, the transcription was entrusted to a specialized professional studio and was always carried out by the same person to ensure that there are no inconsistencies.

As we were interested in the content rather than in the structure of participants' responses for analysis, only long pauses, interruptions, and nonverbal communication (such as laughter) were noted within the text. All transcripts were checked for errors and omissions by listening back to the audio recording and reading the transcripts simultaneously. Each transcript was supplemented with notes made during and immediately after the interview, for example, noting information regarding proxemics and meaningful body language that had been noted down during the interviews and focus groups by one of the researchers observing for this purpose.

### Step 2: Familiarization With the Interview

Members of our research team thoroughly read and re‐read each transcript to become familiar with the whole dataset. We found this familiarization process essential in cases where the researcher analyzing the data had not been present during the interview. We also recorded initial impressions in the margins of transcripts, for example, where participants expressed contrasting views or became emotional. Familiarization through reading and making notes also enabled us later to find our way easily around hundreds of pages of transcript in the analysis.

### Step 3: Coding

Initially, two members of our research team (AC and SC), each from different backgrounds, independently coded the same two transcripts. We underlined with different colors interesting portions of the text and used the margins to describe the content of each passage with a label or code. The underlined portions of the text could range from only a few words to parts of sentences or whole paragraphs. We then used Post‐it notes to record more detailed notes and ideas, for example, questions to bear in mind as the analysis proceeded and ideas for explanations or patterns in the data.

### Step 4: Developing a Working Analytical Framework

After the two researchers had each Open Coded the same two transcripts, a meeting was held to discuss the labels they had assigned to each passage. Working through all of the two transcripts, each coded section was discussed in terms of why it had been interpreted as meaningful, what it told us about participants' views on their “unmet needs,” and how it might be useful for answering the research question. Generally, the same passages of text were highlighted as meaningful by both the reviewers. However, the researchers sometimes interpreted the content in different ways. Those parts of the transcript were therefore reviewed together, and an agreement on which code better captured the idea expressed by the participants was reached. Often, a single code could not capture all the nuances of the patients' words and body language, so the same sentences were labeled in more than one single code.

After discussion, a set of codes was agreed on, each with a brief definition. This formed the initial analytical framework. The same two researchers then independently coded four more transcripts using the initial framework, taking care to note any new codes or impressions that did not fit the existing set. NVivo release 1.7.12020, a qualitative data analysis computer software package produced by Lumivero, was used to speed up the process and to ensure that the data could be easily retrieved at later stages.

Once those transcripts were coded by both of the reviewers, they met again to review the initial framework incorporating new and refined codes through discussion. At this point, they also decided that some codes were conceptually related and therefore should be grouped together. For example, the need to manage aspects related to nutrition and weight control and the need to manage sleep, bladder, and/or intestinal issues were grouped together to make an overarching topic area which was named ‘Need to manage basic body functions’. The process of reviewing, applying, and refining the analytical framework was repeated until no new codes were generated. The final framework consisted of 44 codes, clustered into 16 topic areas, each with a brief explanatory description of its meaning to provide consistency of coding. In Supplemental content 4, an example is provided showing two different topic areas from the final analytical framework with constituent codes and their descriptions.

### Step 5: Applying the Analytical Framework

The final analytical framework was applied to each transcript using NVivo. The two researchers systematically went through each transcript, highlighting each meaningful passage of text and selecting and attaching an appropriate code from the final analytical framework. NVivo was then used to share the indexed transcripts with a third researcher (LG), ensuring that each researcher could access the whole dataset for the next stage of the process. Coding discrepancies were discussed and resolved among the three researchers.

### Step 6: Charting Data onto the Framework Matrix

Once all the data had been coded using the analytical framework, they were summarized in a matrix for each theme using Microsoft Excel. The matrix consisted of one row per code and one column per each patient group based on cancer site. A separate spreadsheet was used for each topic area. Data from transcripts for each participant and code were then abstracted, summarized using the patients' own words, and inserted into the corresponding cell in the matrix. The use of the NVivo software allowed for quick and easy retrieval of indexed data for specific codes within each transcript.

### Step 7: Interpreting the Data

Themes were generated from the dataset by reviewing the matrix and making connections within and between participants and topic areas trying to understand why that particular need had emerged as unmet. This process was guided both by the original research objectives and by new concepts generated inductively from the data. During the interpretation stage we tried to go beyond descriptions of individual cases to develop themes which offered possible explanations for what was happening within the data. Ideas were generated explored and fleshed out through the use of analytical charts and discussion within the team. Supplemental content 3 provides an example of a chart that was written about the topic area ‘Need for support and relationship management’

## Appendix C Analytical Framework Example

An example is provided showing two topic areas from the final analytical framework with constituent codes, their descriptions, and the probable reasons underlying that need based on the participants' words (themes; see Table A1).

**TABLE A1:** Analytical framework example.

Topic area	Code	Description	Themes
Needs related to the management of emotional‐psychological aspects			
	Need to be reassured about one's worries	Holistic aspects related to one's concerns related to being a cancer survivor	Existential vulnerabilty
	Need to manage expectations (one's own and others')	Aspects related to managing one's own expectations and those of others, about one's way of dealing with the disease and the condition of survivorship	Pursuit of control over one's life
	Need for positive thoughts and lightness	Aspects linked to the need to release heavy thoughts and recover a bit of lightness in everyday life	Desire for normality
	Need to rationalize the fear of recurrence	Aspects linked to the need not to be overwhelmed by the fear of relapses and to be able to plan a future	Existential vulnerabilty
	Need to switch off thoughts	Aspects related to freeing the mind from thoughts related to one's condition as a survivor	Existential vulnerabilty
	Need for emotional support	Aspects related to the correct management of one's emotions	Pursuit of control over one's life
Need for support and management of relationships with others			
	Need for support from and relationships with healthcare professionals	Aspects related to the relationship with healthcare professionals, the need to be respected by them and to maintain one's dignity	Perceived dignity and respect
	Need for support from and relationships with partners, children, and family	Aspects linked to the relationship with one's family members, with the search for a newfound normality and respect for one's condition, and the feeling of being in control of one's life	Desire for normality Perceived dignity and respect Pursuit of control over one's life
	Need for support from and relationships with others (friends, coworkers, etc.)	Aspects related to relationships with other people, in search of a newfound normality	Desire for normality

## Appendix D Analytical Chart

An example of an analytical memo that was written about the topic area ‘Need for information’ is provided.

### Definition

Being in possession of all the information needed to be fully in control of one's situation and therapeutic pathway is perceived as very important but difficult to fully achieve. Medical and practical difficulties (e.g., knowing the full range of available services and aids) challenge the utopian ideal of feeling fully in control.

### Codes

Need for more and clearer information about one's current health status; Need for information on available services; Need for more information about medical devices (e.g., for incontinence) and how to use them; Need for more information on how to aid one's recovery; Need for more information on possible therapeutic pathways; Need to receive clear information about personal rights; Need to receive more information about the purpose and side effects of therapies; Need to know who to turn to in case of need.

### Summary of data

- Need for more and clearer information about one's current health status.

## Appendix E Focus Group Details

Table A2

**TABLE A2:** Focus groups details.

Focus groups			
FG	Participants	Cancer typology	n°
1	CSs	Prostate cancer	4
2	CSs	Breast cancer	5
3	CSs	Lymphoma	3
4	CSs	Myeloma	5
5	CSs	Breast	4
6	CSs	Colorectal cancer	5
7	CGs		4
8	CGs		2

Abbreviations: CGs, Caregivers; CSs, Cancer Survivors; n°, number.
